# Sexual Dysfunction in Traumatic Brain Injury: A Narrative Review and Call for Multidisciplinary Framework

**DOI:** 10.3390/life15111659

**Published:** 2025-10-23

**Authors:** Ioannis Mavroudis, Foivos Petridis, Dimitrios Kazis, Gabriel Dăscălescu, Alin Ciobica, Ciprian Ilea, Sorana Caterina Anton, Emil Anton

**Affiliations:** 1 Department of Neuroscience, Leeds Teaching Hospitals, NHS Trust, Leeds LS1 3EX, UK; 2 Faculty of Medicine, Leeds University, Leeds LS2 9JT, UK; 3 Third Department of Neurology, Aristotle University of Thessaloniki, 54124 Thessaloniki, Greece; 4 Academy of Romanian Scientists, 050044 Bucharest, Romania; 5 Department of Biology, Faculty of Biology, “Alexandru Ioan Cuza” University of Iași, Carol I Avenue, No. 20A, 700505 Iași, Romania; 6 “Ioan Haulica” Institute, Apollonia University, Pacurari Street 11, 700511 Iași, Romania; 7 “Olga Necrasov” Center, Department of Biomedical Research, Romanian Academy, 700506 Iași, Romania; 8 Center of Biomedical Research, Romanian Academy, Bd. Carol I, No. 8, 700505 Iași, Romania; 9 Faculty of Medicine, University of Medicine and Pharmacy “Grigore T. Popa”, 700115 Iași, Romania

**Keywords:** sexual dysfunction, traumatic brain injury, multidisciplinary rehabilitation, hormone replacement therapy, cognitive behavioral therapy, gender-sensitive approaches

## Abstract

Background: Sexual dysfunction (SD) is a common yet under-recognized consequence of traumatic brain injury (TBI), with significant implications for physical health, psychological well-being, interpersonal relationships and social reintegration. Although TBI research has largely focused on cognitive, motor and behavioral outcomes, the impact of SD remains insufficiently addressed in both clinical practice and rehabilitation programs. Objectives: This review aims to synthesize current evidence on the prevalence, mechanisms and management of SD following TBI, while emphasizing the importance of gender-sensitive and multidisciplinary approaches to care. Methods: A narrative review was conducted by searching PubMed, Scopus and Web of Science for English-language articles published between 2000 and 2025 using combinations of the following keywords: traumatic brain injury, sexual dysfunction, neuroendocrine dysfunction, psychological sequelae and rehabilitation. Priority was given to peer-reviewed clinical studies, systematic reviews and expert consensus guidelines that addressed neurological, endocrine, cognitive, psychological and social aspects of SD in TBI survivors. Exclusion criteria included case reports with insufficient clinical detail and non-peer-reviewed sources. Articles were screened for relevance to both pathophysiological mechanisms and therapeutic strategies. Results: The etiology of post-TBI SD is multifactorial, involving direct neurological injury, hypothalamic–pituitary dysfunction, emotional and cognitive impairments, as well as psychological challenges such as stigma and relationship strain. Men and women may present distinct symptom profiles; for instance, men more frequently report erectile dysfunction and hypogonadism, whereas women more commonly experience challenges with arousal, lubrication and psychological stress. Effective interventions include pharmacotherapy, hormone replacement therapy, psychotherapy and rehabilitative approaches designed to restore intimacy and quality of life. Optimal outcomes are achieved through multidisciplinary collaboration among neurology, endocrinology, psychiatry, psychology and rehabilitation medicine. Conclusions: Sexual dysfunction should be recognized as a critical component of TBI sequelae rather than a secondary concern. Routine screening, gender-sensitive assessment and the integration of individualized, multidisciplinary care pathways are essential to improving patient outcomes. Advancing clinical awareness and standardization in this area holds the potential to significantly enhance the holistic recovery and reintegration of TBI survivors.

## 1. Introduction

Sexual dysfunction (SD) represents a significant yet frequently overlooked sequela of traumatic brain injury (TBI) [1]. Globally, TBI affects millions of individuals each year and while rehabilitation efforts largely emphasize physical, cognitive and motor recovery, sexual health, as essential determinant of overall quality of life, remains comparatively neglected [2]. The manifestations of SD after TBI are diverse, encompassing decreased libido, erectile dysfunction, impaired arousal, difficulties with orgasm and, in some cases, inappropriate sexual behaviors (ISB). Importantly, these disturbances affect both men and women and they arise from the intricate interplay among neurological, endocrine, cognitive, psychological and social domains [3].

To provide context for a broader readership, it is pertinent to outline key epidemiological and clinical aspects of TBI. Traumatic brain injury represents a significant global public health challenge, with an estimated incidence of over 50 million new cases annually worldwide. It disproportionately affects young adults, particularly males, with a male-to-female ratio approximating 2:1, though incidence rates vary across regions, ethnicities and socioeconomic strata [4]. Common etiologies include road traffic accidents, falls, occupational hazards, assaults and military-related blasts. The frontal and temporal lobes are among the most frequently injured brain regions due to their proximity to bony cranial protuberances, often resulting in a characteristic profile of cognitive, behavioral and executive dysfunctions. Beyond the sexual dysfunctions that are the focus of this review, common sequelae encompass a wide spectrum, including motor impairments, sensory deficits, cognitive slowing, emotional lability and communication difficulties. Prognosis is highly variable and contingent upon injury severity, while mild TBI (mTBI) often resolves with minimal long-term sequelae, moderate to severe injuries can lead to permanent disability, with mortality rates in severe cases remaining substantial. This heterogeneity underscores the complexity of TBI and the necessity for personalized, multi-faceted rehabilitation approaches [2].

From a neurological perspective, TBI can disrupt brain structures that are essential for regulating sexual function, with the specific deficits varying based on the location and severity of the injury [5]. These regions mediate processes such as hormone secretion, impulse regulation and emotional integration, all of which are critical for maintaining sexual health. Endocrine dysfunction is particularly prevalent post-TBI, with hypopituitarism frequently observed in moderate-to-severe cases. Such impairments often result in reduced production of gonadal hormones, including testosterone and estrogen, thereby contributing to decreased libido and impaired sexual performance [6]

Psychological sequelae further exacerbate sexual dysfunction in TBI survivors. Depression, anxiety and post-traumatic stress disorder (PTSD) are highly prevalent in this population and strongly correlated with reduced sexual desire, erectile difficulties, avoidance of intimacy and lower relationship satisfaction [7]. Cognitive deficits, especially those involving executive functions and impulse control, can additionally result in inappropriate sexual behaviors that disrupt social and intimate relationships.

The consequences of SD extend beyond individual impairment to significantly affect relational and social well-being. Altered sexual behavior can strain intimate partnerships, producing frustration, emotional distress and reduced self-esteem for both patients and their partners. Conversely, the restoration of sexual health is intricately linked to improved psychological outcomes, enhanced relationship satisfaction and an overall better quality of life for TBI survivors [8].

Despite its profound impact, sexual dysfunction following TBI is consistently underdiagnosed and undertreated in clinical practice. Contributing factors include the absence of standardized screening tools, limited awareness among healthcare professionals and discomfort in initiating discussions about sexual health [1]. However, recent research increasingly emphasizes the clinical importance of sexual rehabilitation in TBI survivors, underscoring the urgent need for integration of sexual health into standard post-TBI care [9].

While previous reviews have summarized aspects of post-TBI sexual dysfunction, they often focus on isolated domains (e.g., neurological or psychological). This narrative review aims to synthesize these disparate strands of evidence into a cohesive biopsychosocial framework. A specific gap we address is the pronounced disparity in research and clinical focus between male and female TBI survivors. Therefore, this review critically examines the distinct mechanistic pathways and management needs for men and women. Furthermore, by moving from evidence synthesis to the proposal of practical, implementable multidisciplinary care pathways, this work seeks to bridge the significant gap between research knowledge and its application in clinical rehabilitation settings.

The purpose of this narrative review is therefore threefold: (1) to provide a comprehensive synthesis of the neurobiological, endocrine, cognitive and psychological mechanisms underlying SD in patients with TBI; (2) to highlight gender-specific differences in presentation and treatment needs and (3) to discuss current therapeutic approaches, with a particular focus on pharmacological, psychological and multidisciplinary strategies. From a holistic and gender-sensitive perspective, this review seeks to promote improved recognition, screening and management of sexual dysfunction in TBI rehabilitation, thereby contributing to enhanced long-term outcomes for survivors.

## 2. Materials and Methods

This article was conceived as a narrative review. This format was strategically selected to achieve the primary objective of providing a comprehensive and critical synthesis of a highly heterogeneous body of literature spanning neurology, endocrinology, psychology and rehabilitation medicine. Given the intrinsically multifaceted nature of SD following TBI, a narrative review is the most appropriate methodology to incorporate and harmonize diverse forms of evidence, including clinical cohort studies, conceptual frameworks and expert consensus, into a unified biopsychosocial model. A rigid systematic review, while excellent for answering focused clinical questions, is less suited to this task, as its strict inclusion criteria might overlook the very interdisciplinary connections and nuanced clinical insights that are essential for a holistic understanding of the condition. Furthermore, this format provides the necessary scholarly flexibility to not only aggregate existing evidence but also to perform a critical gap analysis, with a particular emphasis on the under-represented issue of gender-specific disparities and to propose forward-looking, implementable frameworks for multidisciplinary care. Thus, this narrative review aims to bridge the significant gap between compartmentalized research findings and the integrated, patient-centered clinical practice required for optimal patient outcomes.

The literature search was conducted across major biomedical databases, including PubMed/MEDLINE, Scopus, Web of Science and Google Scholar. The search was focused on literature published from the year 2000 onwards to capture modern diagnostic and therapeutic paradigms and was extended until July 2025 to include the most recent evidence. Keywords and Medical Subject Headings (MeSH) terms used in various combinations included: “traumatic brain injury”, “sexual dysfunction”, “neuroendocrine function”, “hypopituitarism”, “psychological sequelae”, “cognitive impairment”, “multidisciplinary rehabilitation”, “hormone replacement therapy” and “cognitive behavioral therapy”. Boolean operators (AND/OR) were applied to maximize search sensitivity and specificity.

Eligible studies were restricted to peer-reviewed articles published in English that provide original data, clinical observations or theoretical insights directly addressing sexual health in TBI populations. Excluded were non-English publications, conference abstracts without accessible full-text, studies not related to TBI-associated sexual dysfunction and papers lacking sufficient methodological detail.

The selection process involved two independent reviewers who screened the titles and abstracts, followed by full-text evaluation of potentially relevant studies. In cases where consensus could not be reached, a third reviewer adjudicated to ensure objectivity and methodological consistency. From each eligible study, data were extracted regarding population characteristics, type of sexual dysfunction described, mechanisms involved (neurological, endocrine, psychological, cognitive or social) and therapeutic strategies proposed or evaluated.

All findings were synthesized narratively, with emphasis or recurrent patterns, gender-specific differences and the implications for clinical management. The choice of narrative review format was intentional, as it enabled the integration of heterogeneous sources of evidence, including clinical cohort studies, case reports and conceptual and theoretical frameworks, while maintaining transparency in the search and selection process. This flexible yet rigorous approach provides a comprehensive overview of the mechanisms and treatment options associated with SD in TBI survivors, supporting its clinical relevance.

Although this review does not adhere to the strict methodology of systematic reviews, particular attention was paid to ensuring rigor, transparency and reproducibility. The explicit description of search strategies, eligibility criteria, reviewer procedures and data synthesis was designed to provide a structured and reliable overview, while maintaining the flexibility necessary to address the heterogeneous and multifaceted nature of sexual dysfunction in TBI survivors.

## 3. Results

The literature reviewed highlights that SD is a frequent yet often underrecognized consequence of TBI. Its presentation spans a wide continuum, from mild dissatisfaction with sexual life to severe impairments in desire, arousal, performance and intimacy [1]. This synthesis of findings underscores that SD following TBI is rarely attributable to a single cause. Instead, it arises from the complex interplay between neurological injury, endocrine dysregulation, psychological distress, cognitive impairments and relational factors. Such complexity highlights the need for an integrated interpretative framework that recognizes both the biological underpinnings and the lived human experience of survivors.

It is important to note that the included studies assessed SD using a variety of methods, including validated psychometric scales (e.g., IIEF, FSFI), clinical interviews and hormone level measurements. It should be acknowledged that psychometric instruments provide an estimation of the risk for sexual dysfunction rather than a definitive clinical diagnosis, and their results can be influenced by various subjective factors. Consequently, the term “sexual dysfunction” in this synthesis encompasses both clinically diagnosed conditions and self-reported symptoms measured on a spectrum of severity.

### 3.1. Neurological and Endocrine Findings

Evidence consistently demonstrates that damage to brain regions regulating sexual functions, most notably the hypothalamic–pituitary axis, frontal lobes, brainstem and limbic system, produces significant sexual impairments [10]. These regions play critical roles in hormone regulation, impulse control, emotional processing and autonomic arousal, all of which are central to sexual health. Their disruption explains the wide spectrum of dysfunctions observed, ranging from hypogonadism and loss of libido to disinhibition or inappropriate sexual behaviors [11].

Beyond the specific brain regions involved, the laterality of the injury may also influence the presentation of SD. Although TBI often causes diffuse damage, focal lesions can yield distinct profiles. Some evidence suggests that right-hemispheric lesions, particularly in the frontal and temporal lobes, are more frequently associated with behavioral disinhibition and inappropriate sexual behaviors (ISB) [5]. This may be linked to the right hemisphere’s dominant role in social cognition and the interpretation of nonverbal emotional cues, the disruption of which can lead to impaired social judgement. Conversely, left-hemispheric damage has been more commonly correlated with secondary hyposexuality, a marked decrease in sexual interest, often mediated by a higher prevalence of depression and catastrophic reactions [7]. These patterns are not absolute but highlight how lateralized deficits can shape the clinical picture. From a management perspective, recognizing this laterality can inform targeted interventions. For instance, patients with right-hemisphere signs may benefit more from behavioral strategies and social skills training for disinhibition, while those with left-hemisphere damage and hyposexuality may require a greater focus on treating underlying mood disorders.

Among these, dysfunction of the hypothalamic–pituitary–gonadal axis emerges as a pivotal mechanism. Interruption of its regulatory loops often results in hypogonadism, with reduced testosterone levels in men and estrogen in women [12]. Clinically, these imbalances translate into decreased libido, erectile dysfunction, impaired arousal and reduced sexual satisfaction. Beyond sex hormones, growth hormone deficiency (GHD) has been identified as a long-term sequela, contributing to fatigue, reduced vitality and diminished sexual interest [13]. Importantly, evidence indicates that hypopituitarism and related hormonal deficits may persist for years after TBI, highlighting the necessity of early screening and sustained endocrinological follow-up [14].

Table 1 provides an overview of the main neurological pathways implicated in sexual function and illustrates how their disruption following TBI contributes to distinct dysfunctions.

Complementing these neuroanatomical mechanisms, Table 2 summarizes the major endocrine alterations and their implications for sexual health. Together, these findings reveal how structural and hormonal disruptions converge to undermine sexual well-being after TBI.

### 3.2. Psychological and Cognitive Outcomes

Psychological disturbances, most notably depression, anxiety and PTSD, were consistently associated with poorer sexual outcomes in TBI survivors [25]. Among these, depression emerged as a particularly strong predictor of sexual dissatisfaction. Beyond its well-documented association with low mood, fatigue and anhedonia, depression after TBI often manifests as diminished libido and reduced sexual responsiveness, both of which amplify strain within intimate relationships [26,27]. In turn, relational discord may exacerbate depressive symptoms, generating a bidirectional cycle of emotional distress and impaired sexual health.

Anxiety represents another significant contributor. In many cases, this takes the form of performance anxiety, where fear of sexual inadequacy or relational rejection undermines intimacy. Such anxiety not only predisposes patients to erectile dysfunction but also fosters avoidance of sexual encounters, thereby perpetuating a vicious cycle of worry, failure and withdrawal [28]. Generalized anxiety and hypervigilance, especially common in those with TBI-related PTSD, further complicate matters by producing physiological arousal that is incompatible with sexual desire, while emotional detachment impedes the development of trust and closeness [29].

Cognitive deficits compound these challenges. Impairments in executive functions such as planning, decision-making and impulse control have been repeatedly linked to inappropriate sexual behaviors (ISB), ranging from socially indiscreet remarks to overt disinhibition [15,30,31]. While such behaviors can be profoundly distressing for families and caregivers, they also serve as clinical indicators of frontal and limbic system dysfunction. Importantly, beyond overt disinhibition, cognitive impairments often diminish the capacity for emotional reciprocity, perspective-taking and the intentional planning of intimate encounters, factors essential to sustaining sexual satisfaction and partner bonding [32].

Taken together, these findings underscore that psychological and cognitive sequelae of TBI are not ancillary to neurological and endocrine pathways but rather integral to the lived reality of sexual dysfunction. Indeed, the literature suggests that effective rehabilitation must simultaneously address mood, anxiety and cognitive control if it is to meaningfully restore intimacy and relational quality in this population.

Table 3 outlines the psychological contributors most consistently linked to post-TBI sexual dysfunction, reinforcing their central role alongside neurological and endocrine pathways.

### 3.3. Gender-Specific Patterns

A consistent finding across the literature is that sexual dysfunction manifests differently in men and women following TBI, reflecting both biological and psychological influences. In men, hypogonadism and low circulating testosterone were among the most frequently reported outcomes, with erectile dysfunction representing the predominant clinical complaint [17]. Interventional studies demonstrated that testosterone replacement therapy (TRT) and phosphodiesterase-5 inhibitors such as sildenafil often restored libido and erectile performance, highlighting the potential for effective medical management in male patients. Nevertheless, these interventions address only part of the problem, as relational strain, depressive symptoms and disinhibition frequently persist even when erectile function is pharmacologically restored [37,38].

In contrast, women were more likely to report difficulties related to arousal, lubrication and overall sexual satisfaction. These challenges were frequently compounded by hormonal dysregulation, including altered estrogen and progesterone dynamics, as well as higher vulnerability to depressive and anxiety symptoms [21,39,40,41]. Importantly, qualitative reports suggest that women may experience greater relational disruption and loss of intimacy, as psychological distress interacts with endocrine alterations to create multifaceted barriers to sexual well-being.

Despite these clinically relevant findings, female sexual dysfunction in the context of TBI remains systematically underrecognized and undertreated. Most large-scale clinical studies have disproportionately focused on male participants, leaving substantial gaps in evidence regarding prevalence, mechanisms and treatment responses in women. Few interventional trials have specifically targeted female populations, and standardized assessment tools for post-TBI female sexual function remain scarce. This gender imbalance not only limits generalizability of current evidence but also perpetuates clinical neglect of women’s sexual health [41,42,43].

Taken together, these findings underscore the urgent need for gender-specific diagnostic frameworks, validated assessment instruments and tailored therapeutic strategies. Addressing these disparities requires moving beyond a “one-size-fits-all” approach to rehabilitation and instead adopting individualized care models that account for the unique biological, psychological and relational contexts of men and women living with TBI.

### 3.4. Therapeutic Approaches and Interventions

The results of the reviewed literature consistently emphasize the efficacy of a multimodal therapeutic strategy for maintaining sexual dysfunction post-TBI. Pharmacological interventions, including TRT, estrogen replacement therapy and phosphodiesterase-5 inhibitors such as sildenafil, demonstrated significant effectiveness in restoring hormonal balance and ameliorating physiological aspects of sexual function [22,44,45,46]. Table 4 provides a summary of these pharmacological strategies, highlighting their mechanisms of action and clinical outcomes. However, these interventions alone were insufficient to fully address the complex psychological and relational dimensions of sexual dysfunction, underscoring the need for integrated approaches.

In parallel, psychological interventions play a pivotal role in addressing cognitive, emotional and relational barriers. Cognitive behavioral therapy (CBT) helps patients identify and restructure maladaptive thought patterns related to sexual performance and intimacy, thereby reducing anxiety and depressive symptoms that often exacerbate sexual dysfunction [52]. Sex therapy and couples counseling provide structured environments for patients and their partners to communicate openly, rebuild intimacy and negotiate sexual expectations in a manner sensitive to the sequelae of TBI [53]. Evidence suggests that such interventions are particularly effective when combined with pharmacological treatment, resulting in more comprehensive improvements in sexual desire, satisfaction and overall relational quality.

Furthermore, it is critical to address the physical barriers to sexual activity that often accompany TBI. Motor impairments such as hemiparesis, spasticity, ataxia or reduced coordination can severely hinder the physical act of intimacy, even when physiological sexual functions like erection or lubrication are preserved [54]. These limitations can affect positioning, mobility and the ability to perform stimulatory acts, leading to frustration and avoidance. Rehabilitation must therefore encompass practical, physical aspects of sexual activity. Occupational and physical therapists can provide invaluable guidance on adaptive techniques, assistive devices and several positioning to overcome motor limitations. The use of specialized cushions, adjustable beds and sex aids can facilitate engagement and enhance autonomy, thereby restoring not only function but also confidence and intimacy for both the survivor and their partner [55].

Beyond pharmacological strategies, a growing body of literature supports the incorporation of multidisciplinary rehabilitation programs. These programs coordinate care across neurology, endocrinology, psychology, urology, gynecology and sex therapy, allowing for simultaneous attention to physiological deficits, hormonal dysregulation, emotional well-being and social functioning [56]. Such integrated models have been associated with higher patient satisfaction, improved adherence to treatment plans and better long-term outcomes, highlighting the importance of collaborative care in TBI-related sexual dysfunction [57].

Furthermore, interventions tailored to gender-specific needs are emerging as critical components of effective management. For men, pharmacological restoration of testosterone and erectile function remains central, but complementary psychological support is necessary to address anxiety, depression and relational concerns [58,59]. For women, interventions frequently require a more nuanced combination of hormonal therapy, psychosexual counseling and strategies targeting arousal and intimacy difficulties, reflecting the complex interplay between endocrine, cognitive and emotional factors [60,61].

Collectively, these findings reinforce the notion that sexual dysfunction post-TBI is not a single-dimensional problem amenable to isolated treatment. Optimal outcomes are achieved when therapeutic interventions are personalized, multimodal and sensitive to both biological and psychosocial determinants, ensuring that recovery encompasses both physiological restoration and the enhancement of sexual well-being and quality of life.

### 3.5. Multidisciplinary Integration

While principles of multidisciplinary care are supported by broader rehabilitation literature, a growing body of evidence specific to TBI underscores its value for sexual dysfunction [62,63]. Collaborative approaches that integrate the expertise of neurologists, endocrinologists, psychologists, urologists, gynecologists and sex therapists have been associated with the most comprehensive and sustained improvements in sexual health and overall quality of life [63]. By combining medical, psychological and relational interventions, these teams are able to provide individualized care, addressing both the biological underpinnings of sexual dysfunctions, such as hormonal imbalances, neurological deficits and pharmacological needs and the psychosocial consequences, including anxiety, depression, relational strain and impaired intimacy.

Multidisciplinary care also facilitates coordinated assessment and follow-up, ensuring that interventions are continuously adjusted based on patient progress [64]. For example, endocrine dysfunctions can be closely monitored alongside psychological response to therapy, allowing for timely modification of hormone replacement regimens or psychotherapeutic approaches. Similarly, neurologists and sex therapists can jointly address the consequences of cognitive or behavioral impairments on sexual function, developing strategies that are both physiologically and socially appropriate [65].

Importantly, patient-centered communication within multidisciplinary teams encourages active participation and empowerment. Patients and their partners are more likely to engage in treatment plans when they perceive that care is coordinated, holistic and sensitive to their experiences. Such engagement has been linked to better adherence to pharmacological interventions, greater uptake of psychotherapeutic strategies and improved satisfaction with sexual and relational outcomes [66].

Evidence further suggests that multidisciplinary programs improve long-term rehabilitation outcomes by integrating sexual health into broader TBI recovery strategies [67]. Rather than treating sexual dysfunction as an isolated symptom, these approaches embed interventions within the context of overall physical, cognitive and emotional rehabilitation, emphasizing the interdependence of these domains in promoting well-being [68].

In summary, multidisciplinary integration represents a best-practice standard for managing sexual dysfunction after TBI, ensuring that interventions are comprehensive, personalized and responsive to the complex interplay of biological, psychological and social determinants [3,53]. The success of such programs underscores the necessity of collaborative care models in both clinical and research contexts, highlighting the need for ongoing education. Interprofessional coordination and systematic evaluation of patient outcomes.

### 3.6. Overarching Patterns and Gaps

The synthesis of the reviewed literature highlights several consistent patterns regarding sexual dysfunction in TBI survivors. First, the etiology is clearly multifactorial, with neurological injury, endocrine dysregulation and psychological disturbances interacting to produce a spectrum of sexual impairments. This complexity underscores the need for a holistic, patient-centered approach rather than isolated interventions [2,69].

Second, gender-specific differences in presentation are evident, influenced by both biological and psychosocial factors. Men more frequently report difficulties rooted in hypogonadism and erectile function, while women more often report challenges with arousal, lubrication and a greater interplay with psychological distress. It is critical to note that these are predominant patterns and significant overlap exists, for example, men experience psychological sequelae and women can suffer from hormonal imbalances. These differences emphasize the importance of incorporating gender-specific assessment and intervention strategies into clinical practice to ensure tailored and effective care [45,47,70].

Third, therapeutic approaches show differential effectiveness depending on the dimension of dysfunction addressed. Pharmacological interventions, such as TRT, estrogen therapy and phosphodiesterase-5 inhibitors, reliably improve physiological deficits [23,24,71,72]. However, maximal benefit requires the addition of psychological and behavioral interventions, including cognitive behavioral therapy, sex therapy and couples counseling, to address emotional, cognitive and relational barriers [16].

Despite these insights, the literature reveals significant gaps and limitations. Research on female sexual dysfunction post-TBI is sparse, with few studies providing dedicated evaluation of gender-specific treatment modalities. Long-term outcomes of both pharmacological and psychological interventions are insufficiently characterized, and the evidence base is constrained by studies with limited sample size (often <50), which reduces statistical power and generalizability. Moreover, standardized screening protocols for sexual dysfunction in TBI survivors remain largely absent, contributing to underdiagnosis and inconsistent clinical management [43,73].

Taken together, these observations point to a critical need for well-designed, longitudinal studies, incorporation of standardized assessment tools and development of integrated, multidisciplinary care pathways. Addressing these gaps is essential not only to improve sexual health outcomes but also to enhance overall quality of life and rehabilitation success in TBI survivors.

## 4. Discussion

This review synthesizes evidence confirming that SD following TBI is a complex, multifactorial condition that transcends mere physiological disruptions. The findings compellingly demonstrate that sexual health is not a peripheral concern but a central determinant of quality of life, intricately woven into the fabric of neurological, endocrine, cognitive, psychological and social recovery. The critical insight that emerges is the profound interdependence of these domains. For instance, a neurological injury causes hormonal imbalance, which exacerbates depression, which in turn strains relationships, creating a self-perpetuating cycle of dysfunction. Therefore, the clinical imperative is to move beyond siloed interventions and embrace integrative care models.

### 4.1. Neurobiological Underpinnings and Endocrine Dysregulation

The neurobiological foundations of SD following TBI are profound and multifaceted. Traumatic injury to brain regions such as the hypothalamic–pituitary axis, frontal lobes, brainstem and limbic system initiates a cascade of structural and functional disruptions that reverberate throughout sexual, emotional and relational domains [18,74].

Among these, the hypothalamic–pituitary–gonadal axis emerges as a crucial hub. Even subtle injuries to this axis can impair gonadotropin secretion, leading to hypogonadism and a reduction in circulating testosterone in men and estrogen in women [75,76]. Clinically, these deficits manifest as hypoactive sexual desire, erectile dysfunction and reduced capacity for arousal and lubrication. Unlike transient hormonal changes, post-TBI hypogonadism frequently persists for years, suggesting a chronic impairment rather than an adaptive recovery process. This persistence highlights the need for long-term endocrine surveillance, as patients may remain symptomatic despite improvements in other areas of rehabilitation [3,19,20].

The role of GHD further underscores the systemic impact of TBI. Beyond its well-documented effects on somatic growth and metabolism, growth hormone contributes significantly to energy, vitality and overall quality of life. Its deficiency often translates into fatigue, diminished stamina and decreased sexual motivation [77,78]. Survivors may describe a profound “flattening” of sexual interest, which is not merely psychological but rooted in impaired neuroendocrine signaling.

What becomes evident from the literature is that neuroendocrine dysfunction cannot be interpreted in isolation. Hormonal disturbances amplify psychological vulnerability: testosterone deficiency, for example, is strongly linked not only with reduced libido but also with depressive symptoms, irritability and relational withdrawal. Similarly, estrogen deficits in women intensify arousal difficulties while also contributing to mood instability and cognitive fog. Thus, the neurobiological and endocrine pathways intertwine with psychological dimensions, creating a self-reinforcing cycle of dysfunction [49].

Another crucial aspect is the temporal dimension of endocrine dysfunction. While acute disruptions may occur in the immediate aftermath of TBI, many patients exhibit delayed-onset hypopituitarism, only recognized months or even years later [72,79]. This latency complicates clinical recognition and underlines why systematic, longitudinal follow-up is essential. Unfortunately, routine post-TBI care often neglects endocrine evaluation, allowing SD to remain unaddressed despite being biologically identifiable and clinically treatable.

From a therapeutic standpoint, the recognition of these mechanisms opens avenues for targeted interventions. TRT in men and estrogen replacement in women can significantly improve sexual desire, arousal and overall satisfaction [33,80]. However, endocrine therapy alone cannot resolve the broader psychosocial and relational consequences of SD. Hormonal restoration must therefore be seen as a necessary but insufficient step, embedded within a broader multidisciplinary rehabilitation strategy.

Therefore, a proactive and sustained endocrinological assessment is a critical, yet often missing, component of post-TBI care, forming the biological cornerstone upon which broader rehabilitative efforts must be built.

### 4.2. Psychological and Cognitive Contributions

While the biological and endocrine dimensions of post-TBI SD are well established, the psychological and cognitive sequelae often prove equally, if not more, debilitating. Evidence consistently demonstrates that mood disturbances, anxiety states, PTSD and cognitive impairments exert a profound influence on survivors’ sexual health and intimate relationships [25,51]. These disturbances are not peripheral, but rather central to the lived experience of SD, shaping desire, satisfaction and relational stability.

Depression emerges as a particularly potent predictor of diminished sexual functioning. Beyond its classic manifestations of low mood, anhedonia and fatigue, depression directly erodes libido and intensifies relational strain [34,81]. Survivors often report a loss of sexual initiative, reduced responsiveness to intimacy and feelings of inadequacy, which in turn perpetuate a cycle of withdrawal and dissatisfaction. Importantly, this depressive burden cannot be dismissed as a mere psychological reaction to disability; rather, it interacts dynamically with neuroendocrine deficits (e.g., hypogonadism) and cognitive limitations, amplifying their impact [3,5,82].

Anxiety disorders, including generalized anxiety and performance anxiety, also exert a destabilizing influence. Heightened arousal of the autonomic nervous system, intrusive worries about sexual performance and hypervigilance often culminate in erectile dysfunction or avoidance of intimacy altogether [35,83,84]. In many cases, anxiety undermines the confidence necessary for sexual engagement, creating a feedback loop where fear of failure generates actual dysfunction, further consolidating avoidance patterns. Such dynamics not only compromise sexual health but also erode self-esteem and relational cohesion.

The impact of PTSD is particularly profound in survivors of TBI sustained in traumatic contexts, such as accidents, assaults or combat exposure. Emotional numbing, intrusive recollections and hyperarousal frequently hinder the capacity for intimacy. For many patients, sexual encounters may trigger intrusive memories or exacerbate hypervigilance, leading to avoidance of intimacy or reduced desire. Partners often misinterpret this withdrawal as rejection, adding relational strain to an already fragile recovery trajectory [36,85].

Disturbances in body image represent another significant psychological sequela with direct implications for sexual quality of life. TBI can result in visible scars, paralysis, weight changes or use of assistive devices, all of which can alter a person’s self-perception and sense of attractiveness. This dissonance between pre-injury self-image and post-injury reality can evoke feelings of shame, unattractiveness and sexual undesirability, leading to social withdrawal and avoidance of intimate situations [85]. The internalization of a “damaged” identity can be as debilitating as any physical limitation, directly corroding sexual confidence. Addressing these concerns through targeted psychotherapeutic interventions, such as body image-focused cognitive behavioral therapy and supportive counseling, is essential for helping survivors reclaim their sexual self-esteem and reengage with intimacy [20].

Alongside these psychiatric dimensions, cognitive impairments represent another critical determinant of post-TBI sexual dysfunction. Deficits in executive functions, planning, decision-making, impulse control and self-monitoring frequently manifest as ISB [86]. These behaviors, ranging from disinhibition to socially inappropriate advances, are often distressing for families and caregivers, highlighting the profound role of frontal lobe and limbic dysfunction in regulating sexual conduct. Beyond overt disinhibition, subtle cognitive deficits also reduce the capacity for emotional reciprocity, sexual planning and sustained engagement in intimacy. Survivors may struggle to interpret social cues, maintain emotional attunement or sustain sexual interest, resulting in diminished relational satisfaction.

What becomes evident is that psychological and cognitive dimensions rarely act in isolation. Rather, they intertwine with biological disturbances to form a multifactorial matrix of dysfunction. For instance, hypogonadism may reduce libido, while concomitant depression deepens disinterest and executive dysfunction prevents adaptive coping, culminating in a compounded burden far greater than the sum of its parts [87].

From a clinical perspective, this complexity demands careful assessment. Screening for depression, anxiety, PTSD and cognitive deficits should be an integral component of post-TBI care, not an afterthought. Equally, interventions cannot be limited to symptom management alone. CBT, trauma-focused psychotherapy and structured cognitive rehabilitation have shown promise for mitigating the psychological and cognitive barriers to intimacy. When combined with pharmacological or hormonal strategies, these interventions enable a more comprehensive restoration of sexual health [48].

The cumulative effect of these physiological, cognitive and physical changes often places immense strain on intimate partnership. Sexual dysfunction is a frequently cited contributor to relational discord, with partners reporting feelings of rejection, loss of intimacy and caregiver burden that can evolve into chronic conflict. Evidence suggests that the rate of relationship breakdown, including separation and divorce, is significantly higher among TBI survivors compared to the general population, with sexual issues being a pivotal factor [88]. The partner’s well-being is thus intrinsically linked to the rehabilitation process. Interventions must be couple-centric, focusing on rebuilding trust, fostering open communication about changes in sexual needs and abilities and redefining intimacy in ways that are mutually satisfying. Proactive couples counseling is not merely beneficial but often essential for preserving the partnership, which itself is a critical support system for the survivor’s long-term recovery [8].

Consequently, the management of post-TBI SD is incomplete without rigorously addressing the psychological and cognitive landscape of the survivor, necessitating integrated screening and therapy that targets mood, trauma, self-image and executive function within the context of the couple’s relationship.

### 4.3. Gender—Specific Considerations

One of the most compelling findings of the reviewed literature is the clear divergence in how SD manifests and is experienced by men and women after TBI. While overarching mechanisms are shared, their clinical expression and impact on sexual health are markedly gendered. This divergence underscores the necessity of gender-sensitive assessment and intervention strategies, which remain underdeveloped in current clinical practice [1,2,89].

In male patients, SD is often dominated by biological and hormonal disturbances. Hypogonadism, frequently arising from hypothalamic–pituitary axis disruption, results in reduced testosterone levels and is strongly correlated with decreased libido, erectile dysfunction and impaired sexual performance [90]. The availability of relatively well-established treatments, such as TRT and phosphodiesterase type 5 inhibitors like sildenafil, means that male dysfunction is more frequently recognized and treated in clinical settings. Evidence suggests that these interventions can effectively restore aspects of sexual function, through their success depends on concurrent management of psychological comorbidities such as depression or anxiety. Nevertheless, even in men, therapeutic outcomes are variable, and many continue to report residual dissatisfaction despite pharmacological intervention [44,50].

In female patients, the picture is notably different and considerably more complex. Women with TBI are more likely to report impairments in arousal, lubrication, orgasmic capacity and overall sexual satisfaction [21]. Unlike men, where hormonal hypogonadism is a prominent and easily measurable deficit, female sexual dysfunction is frequently underpinned by a broader interplay of psychological distress, relational strain and subtler endocrine changes (e.g., estrogen imbalance). Moreover, the subjective nature of female sexual response, often dependent on emotional attunement, intimacy and psychological well-being, means that depression, PTSD and anxiety exert a disproportionate burden on women’s sexual health. Unfortunately, this complexity has contributed to an underrecognition of female-specific dysfunction in both research and clinical care, as standardized tools often fail to capture the nuances of female sexual experience [91].

The gender gap in research and treatment is striking. Most interventional studies disproportionately focus on men, especially regarding pharmacological strategies. In contrast, women remain underrepresented in clinical trials, and therapeutic approaches tailored specifically to their needs are scarce. This imbalance reflects a broader issue in sexual medicine, where male dysfunction has historically received greater attention, leaving female patients underserved. As a result, many women with TBI-released SD remain undiagnosed and untreated, despite the profound impact these difficulties exert on their quality of life and relationships.

From a psychological perspective, gender differences also extend to how sexual dysfunction is experienced and expressed. Men may be more likely to report frustration linked to performance and erectile capacity, while women may internalize dysfunction as relational strain loss of intimacy or diminished self-worth. Cultural norms and expectations further influence disclosure, with women often less likely to openly report sexual concerns. These factors collectively contribute to a cycle of silence and underrecognition, perpetuating disparities in care.

Taken together, these findings highlight an urgent need for gender-sensitive assessment tools and interventions. Screening instruments must be validated separately for men and women, and treatment strategies must move beyond a “one-size-fits-all” paradigm. For men, pharmacological interventions remain essential but are most effective when combined with psychological support. For women, a stronger emphasis on integrated psychosocial and relational therapies, potentially combined with hormonal modulation, appears critical. Equally important is the inclusion of women in future research, ensuring that clinical recommendations reflect the lived realities of both sexes.

These profound gender differences necessitate a fundamental shift in clinical practice. The following discussion of therapeutic implications must, therefore, be viewed through this essential gender-informed lens.

### 4.4. Therapeutic Implications and Clinical Management

The management of sexual dysfunction (SD) following TBI requires a therapeutic framework that is as multifaceted as the condition itself. Evidence from the reviewed literature strongly suggests that no single intervention is sufficient in isolation. Instead, optimal outcomes are achieved through an integrated, multimodal approach that acknowledges the convergence of neurological, endocrine, psychological and social determinants of sexual health [1,92,93].

Pharmacological therapies remain a cornerstone of management, particularly in addressing endocrine-related dysfunctions. TRT has demonstrated efficacy in restoring libido and improving sexual performance in male patients with hypogonadism [79]. Similarly, estrogen replacement therapy, although less frequently studied in TBI contexts, has shown potential in ameliorating arousal and satisfaction in women experiencing hormonal imbalance [21,94]. Phosphodiesterase-5 inhibitors such as sildenafil provide targeted benefits for erectile dysfunction, restoring physiological arousal mechanisms. However, while pharmacological agents can address the biological substrates of dysfunction, they are insufficient in isolation, as they do not tackle the psychological distress, relational difficulties or cognitive barriers that frequently accompany TBI-related SD [95,96].

Psychological and behavioral interventions, therefore, play an indispensable role in clinical management. CBT has shown promise in reducing maladaptive thought patterns and anxiety surrounding sexual performance, thereby improving confidence and relational intimacy [52]. Sex therapy and couples counseling extend these benefits by addressing communication deficits, relational strain and the psychosocial dimensions of intimacy. Evidence indicates that patients engaging in combined pharmacological and psychological therapy report significantly higher satisfaction compared to those receiving single-modality interventions. Importantly, these therapeutic approaches empower patients and their partners by normalizing conversations around sexuality, reducing stigma and fostering mutual understanding [97,98].

Rehabilitative strategies must also extend beyond individual-level treatments. Incorporating sexual health into the broader rehabilitation framework acknowledges that sexuality is not an ancillary concern but a central determinant of quality of life. Clinicians are encouraged to integrate routine screening for sexual dysfunction into post-TBI follow-up visits, using validated tools to identify issues early. Moreover, rehabilitation teams must recognize the diversity of presentations: for example, inappropriate sexual behaviors stemming from frontal lobe dysfunction require different strategies than diminished libido due to endocrine disruption. Tailored rehabilitation plans, sensitive to these distinctions, enhance both patient well-being and caregiver support [99,100].

Another critical implication relates to the training and awareness of healthcare professionals. Studies reveal that many clinicians lack the confidence or training to initiate discussions about sexual health with TBI patients, leading to underdiagnosis and undertreatment. Normalizing sexual health as part of routine neurological and rehabilitative care is essential. Training programs that equip providers with communication skills and evidence-based intervention strategies are likely to improve recognition and treatment rates [7,101].

A fundamental barrier to effective management is the mutual discomfort in discussing sexual health. Patients often hesitate to report symptoms due to embarrassment, fear of stigmatization or the misconception that sexual function is a low priority in recovery. Mirroring this, many clinicians, including neurologists and rehabilitation specialists, report a lack of confidence, training and time to initiate these conversations, leading to systematic underdiagnosis [101]. This communication gap is compounded by the absence of structured sexual health screening in standard TBI care pathways, is a critical obstacle. Overcoming this requires a paradigm shift towards normalizing sexual health as an integral component of holistic recovery. We advocate for the implementation of brief, validated screening tools during routine follow-ups and the incorporation of communication skills training into medical and rehabilitation curricula. Creating a clinical environment of trust, confidentiality and non-judgmental openness is paramount to empowering patients to voice their concerns [6].

Finally, effective clinical management must adopt a patient-centered orientation. Interventions should be adapted to individuals’ needs, preferences, cultural backgrounds and gender-specific concerns. For some patients, restoring erectile capacity may be the priority, for others, rebuilding intimacy and relational confidence may take precedence. Recognizing this heterogeneity ensures that treatment is not merely symptom-focused but genuinely restorative of the patient’s overall quality of life.

In summary, therapeutic management of post-TBI SD is best understood as a continuum, integrating pharmacological, psychological and relational care within a rehabilitative and patient-centered framework. Moving forward, the challenge lies not only in refining individual treatments but also in embedding sexual health into the standard of care for TBI survivors, ensuring that recovery encompasses not just survival and functional independence, but also the restoration of intimacy and human connection.

### 4.5. Multidisciplinary Integration

The complex and multifactorial nature of SD following TBI necessitates a coordinated, multidisciplinary approach. The literature consistently underscores that optimal outcomes are achieved when care integrates neurological, endocrine, psychological and sexual health expertise [100,102]. Neurologists play a central role in assessing the extent and location of brain injury, identifying the neural circuits implicated in sexual functioning and monitoring the evolution of neurological sequelae [102]. Endocrinologists complement this perspective by detecting hormonal imbalances, such as hypogonadism and growth hormone deficiency, and guiding appropriate replacement therapies [6].

Emerging models in clinical practice demonstrate the successful implementation of such multidisciplinary networks. The INTIMASY-TBI guideline, for instance, was developed through international consensus and provides a structured framework for optimizing intimacy and sexuality by coordinating inputs from neurology, psychology and sex therapy [53]. Another exemplar is documented by Iftekhar et al. (2025), who described a multidisciplinary sexual dysfunction program where patient feedback was actively integrated to refine care coordination among endocrinologists, urologists, gynecologists and mental health professionals, resulting in significantly improved patient-reported outcomes and satisfaction [63]. These models validate that proactive, structured collaboration not only is feasible but also directly enhances the efficacy and patient-centeredness of rehabilitation for post-TBI SD.

Equally crucial is the involvement of psychologists, sex therapists and psychiatrists, who address the cognitive, emotional and relational dimensions of SD. CBT, sex therapy and couples counseling have been repeatedly demonstrated to improve sexual functioning when combined with pharmacological interventions, highlighting the need for integrated care plans [103]. Urologists and gynecologists contribute by managing physiological impairments, such as erectile dysfunctions or lubrication difficulties, thereby ensuring that both the biological and psychosocial aspects of sexual health are addressed [104,105].

Importantly, the multidisciplinary model allows for individualized, patient-centered treatment. By coordinating interventions across specialties, clinicians can tailor therapy to each patient’s specific constellation of deficits, psychological state and relational context. This holistic approach not only addresses the direct manifestations of SD but also mitigates secondary consequences, such as relational strain, diminished quality of life and psychological distress. Overall, the evidence supports that multidisciplinary care is not merely beneficial but essential for meaningful and sustained improvements in sexual health post-TBI.

### 4.6. Resource Allocation and Cross-Cultural Considerations

The prioritization of SD in TBI care is inevitably influenced by broader healthcare resource allocation and socio-cultural contexts. In resource-limited settings, where the focus is often on acute life-saving interventions and basic physical rehabilitation, sexual health is frequently deprioritized [106]. Furthermore, cultural, religious and ethnic norms profoundly shape attitudes toward sexuality, influencing both patients’ willingness to seek help and clinicians’ comfort in providing it. In some cultures, discussing sexuality may be highly taboo, particularly for women and sexual minorities, thereby exacerbating underreporting and isolation [43]. To improve accessibility, innovative strategies are needed. These may include the development of low-cost, culturally adapted educational materials, telehealth consultations to bridge geographical gaps and task-shifting certain counseling roles to trained community health workers. At a policy level, advocating for the recognition of sexual health as a fundamental component of quality of life, as underscored by the World Health Organization, is crucial to legitimizing its place in rehabilitation standards and funding allocation across diverse health systems [107].

### 4.7. Reproductive Concerns, Fertility Preservation and Insights from Spinal Cord Injury

For TBI survivors of reproductive age, sexual dysfunction raises significant concerns regarding fertility and the potential for biological parenthood. Post-TBI hypogonadotropic hypogonadism can directly impair reproductive capacity, yet these issues are frequently overlooked. The clinical approach to fertility preservation and restoration differs fundamentally between male and female survivors.

In male patients, the impact on fertility is a confluence of several distinct issues:

- Sexual Function: Erectile dysfunction and impaired ejaculation present a mechanical barrier to conception.
- Spermatogenesis: Hypogonadotropic hypogonadism results in inadequate testicular stimulation, leading to impaired sperm production (oligo- or azoospermia) [6,19].
- Pre-testicular (Pituitary) Dysfunction: The root cause is the disrupted secretion of gonadotropins (LH and FSH) from the pituitary gland.

Clinical management must address each level. Phosphodiesterase-5 inhibitors can mitigate erectile dysfunction. To obtain semen for fertility purposes, techniques well-established in spinal cord injury (SCI) are applicable, such as penile vibratory stimulation and electroejaculation [108]. If these methods fail, surgical sperm retrieval (e.g., testicular sperm extraction) is an option. Crucially, sperm cryopreservation should be offered early. For men with persistent hypogonadism wishing to restore fertility, gonadotropin replacement therapy can be employed to stimulate spermatogenesis, analogous to protocols used for other etiologies of hypogonadotropic hypogonadism [109].

In female survivors, hypogonadotropic hypogonadism disrupts the hypothalamic–pituitary–ovarian axis, leading to anovulation, menstrual irregularities and amenorrhea [21,22]. This creates a direct physiological barrier to conception. While hormone replacement therapy with estrogen and progesterone can alleviate symptoms of SD (e.g., arousal and lubrication difficulties), it does not restore fertility. For women desiring conception, ovulation induction using exogenous gonadotropin is required. Furthermore, fertility preservation via oocyte or embryo cryopreservation following ovarian stimulation should be discussed, particularly for women whose long-term recovery may complicate future family planning.

A proactive approach is paramount. For all TBI survivors of reproductive age with confirmed or suspected hypogonadism, early referral to a reproductive endocrinology and infertility specialist is strongly recommended [6,72]. This ensures that the opportunity for biological parenthood is integrated into comprehensive, patient-centered care. Future research must directly investigate fertility outcomes and the efficacy of these interventions in the TBI population.

### 4.8. Limitations and Future Directions

While this review synthesizes a wide range of evidence regarding sexual dysfunction after TBI, several limitations should be acknowledged. First, many studies are limited by small sample sizes, single-center design or retrospective methodologies, which may reduce the generalizability of findings. Second, female sexual dysfunction remains underrepresented, with few studies adequately powered to evaluate gender-specific outcomes or treatment strategies. Third, variability in diagnostic criteria, assessment tools and follow-up duration complicates direct comparison across studies, highlighting the need for standardized screening protocols.

Furthermore, this review primarily addressed adult population with moderate to severe TBI. Significant knowledge gaps remain regarding sexual dysfunction in understudied groups, including those with mTBI, pediatric and adolescent survivors, older adults and LGBTQ+ individuals. The manifestations, assessment and management of SD in these diverse populations are likely distinct and warrant dedicated investigation in future research.

Additionally, the restriction of our search to English-language publications may have introduced a selection bias, potentially omitting relevant studies published in other languages.

Future research should prioritize large-scale, multicenter perspective studies to capture the heterogeneity of TBI populations and provide robust evidence for intervention efficacy. Gender-focused investigations are urgently needed to elucidate the differential mechanisms and treatment responses in women. Additionally, long-term follow-up studies are required to assess the durability of both pharmacological and psychological interventions. The integration of patient-reported outcomes and qualitative assessments can further enhance understanding of the lived experience of SD, guiding more patient-centered therapeutic approaches. Finally, the development of comprehensive clinical guidelines that combine neurological, endocrine, psychological and sexual health perspectives will be instrumental in standardizing care and improving the quality of life for TBI survivors.

## 5. Conclusions

SD is a prevalent and multifaceted consequence of TBI, arising from a complex interplay of neurological damage, endocrine dysregulation and psychological and cognitive sequelae. This review underscores that effective management cannot be achieved through isolated interventions. A patient-centered, multimodal and multidisciplinary approach is essential. Crucially, care must be gender-sensitive to address the divergent experiences of men and women. Future efforts must focus on developing standardized screening tools, advancing gender-specific research—particularly for women—and integrating sexual health as a core component of TBI rehabilitation standards. Ultimately, restoring sexual well-being is fundamental to achieving holistic recovery and improving the overall quality of life for TBI survivors.

## Figures and Tables

**Table 1:** Neurological Pathways Affected by TBI and Their Role in Sexual Function.

Brain Region	Role in Sexual Function	Impact of TBI on Sexual Function
Hypothalamic–Pituitary Axis	Regulates sex hormones like testosterone, estrogen [10,12,14]	Impaired libido, hypogonadism
Frontal Lobes	Impulse control, social appropriateness, planning [5,11,15]	Disinhibition or sexual apathy
Limbic System	Emotional regulation, sexual motivation, reward [10,11]	Reduced libido, emotional instability
Brainstem	Controls autonomic arousal and physical responses [10,16]	Impaired erectile function, reduced physiological arousal

**Table 2:** Endocrine Disruptions and Their Sexual Impacts.

Hormonal Dysfunction	Mechanism	Sexual Impact
Hypogonadism (Low Testosterone)	Disruption of HPG axis, reduced production [6,12,17,18]	Loss of libido, erectile dysfunction, impaired sexual performance
Growth Hormone Deficiency (GHD)	Damage to pituitary somatotroph cells [13,19,20]	Fatigue, reduced vitality, decreased sexual interest
Estrogen/Progesterone Imbalance	Disruption of HPG axis in females [21,22,23]	Reduced sexual desire, difficulty with arousal and lubrication, poor satisfaction
Hyperprolactinemia	Disinhibition of pituitary lactotrophs [6,24]	Suppressed libido, hypogonadism via inhibition of GnRH

**Table 3:** Psychological Factors and Their Role in Sexual Dysfunction.

Psychological Factor	Impact on TBI Patients	Effect on Sexual Function
Depression	Low mood, fatigue, irritability [7,25,26,33]	Reduced libido, poor sexual satisfaction, relational withdrawal
Anxiety & PTSD	Hypervigilance, performance anxiety, emotional numbing [7,28,29,34,35]	Erectile dysfunction, avoidance of intimacy, reduced desire
Cognitive Impairment (Executive Dysfunction)	Poor impulse control, impaired planning, reduced self-monitoring [5,15,31,36]	Inappropriate sexual behaviors, difficulty with sexual planning and reciprocity
Altered Self-Image & Stigma	Distress over changed identity and capabilities [8,18,32]	Reduced sexual confidence, avoidance of intimate situations

**Table 4:** Pharmacological Treatments for Sexual Dysfunction Post-TBI.

Medication	Indication	Mechanism of Action	Effectiveness in TBI Patients
Sildenafil (Viagra)	Erectile Dysfunction	Enhances nitric oxide-mediated vasodilatation in penile tissue [44,47,48]	Effective for improving erectile function; evidence primarily for male TBI populations
Testosterone Replacement Therapy	Hypogonadism	Restores physiological testosterone levels [37,44,49]	Improves libido and sexual performance; monitoring of PSA and hematocrit required
Estrogen/Progesterone Therapy	Female Hormonal Deficiency	Restores physiological estrogen/progesterone levels [21,22,50]	Can improve arousal, lubrication and satisfaction; evidence in TBI is limited
Antidepressants (SSRIs)	Depression-related SD	Modulates serotonin levels [33,51]	May improve mood, but often have side effects that worsen sexual function (e.g., delayed orgasm)

## Data Availability

No new data were created in this study.
